# Aging and spatial cues influence the updating of navigational memories

**DOI:** 10.1038/s41598-019-47971-2

**Published:** 2019-08-07

**Authors:** Maayan Merhav, Thomas Wolbers

**Affiliations:** 1 German Center for Neurodegenerative Diseases (DZNE), Aging and Cognition Research Group, Leipziger Str. 44, 39120 Magdeburg, Germany; 2 Center for Behavioral Brain Sciences, Brennecke Str. 6, 39118 Magdeburg, Germany; 3 Faculty of Medicine, Otto-von-Guericke-University Magdeburg, Leipziger Str. 44, 39120 Magdeburg, Germany

**Keywords:** Cognitive neuroscience, Human behaviour, Cognitive ageing

## Abstract

Updating navigational memories is important for everyday tasks. It was recently found that older adults are impaired in updating spatial representations in small, bi-dimensional layouts. Because performance in small-scale areas cannot predict navigational behavior, we investigated how aging affects the updating of navigational memories encoded in large, 3-dimensional environments. Moreover, since locations can be encoded relative to the observer (egocentric encoding) or relative to landmarks (allocentric encoding), we tested whether the presumed age-related spatial updating deficit depends on the available spatial cues. By combining whole-body motion tracking with immersive virtual reality, we could dissociate egocentric and allocentric spatial cues and assess navigational memory under ecologically valid conditions (i.e., providing body-based and visual cues). In the task, objects were relocated overnight, and young and older participants had to navigate to the updated locations of the objects. In addition to replicating age-related deficits in allocentric memory, we found age-related impairments in updating navigational memories following egocentric encoding. Finally, older participants depicted stronger representations of the previous navigational context that were correlated with their spatial updating deficits. Given that these effects may stem from inefficient suppression of former navigational memories, our findings propose a mechanism that helps explain the navigational decline in aging.

## Introduction

Navigational abilities decline with aging^1–3^, but the underlying mechanisms are not fully understood^2^. In the current study, we aimed to test for a mechanism that can contribute to the navigational deficits in old age. Specifically, we hypothesized that older adults are impaired in their ability to *update* long-term navigational memories. The ability to update long-term, navigational information is required in many everyday situations – for example, when looking for a car in a frequently visited parking lot, one has to retrieve today’s parking place over yesterday’s location. Studies in rodents have shown that the ability to update long-term spatial representations is impaired in old age^4,5^. Specifically, in the aging hippocampus, place cell firing patterns associated with a previously learned environment are often abnormally maintained in a novel environment^5^, but corresponding evidence in humans is still missing.

Human aging is known to affect path integration, the ability to update one’s location during self-motion^6–9^. In those studies, however, the updating process operated on transient working memory representations over periods of seconds. In contrast, in the current study, we explored the ability to update long-term navigational representations over a 24 hours period. Recent findings have demonstrated age-related deficits in the ability to update long-term, spatial representations on a small-scale, bi-dimensional layout^10^. In that study, older adults showed particular difficulties in retrieving locations of objects – presented on a computer screen – that were relocated overnight. However, spatial processing of small, bi-dimensional layouts is not identical to navigation in real environments, because the latter also involves bodily movements of the observer, perception of 3D spatial information, etc.^11^. Thus, it remains to be determined whether older adults are also impaired in updating long-term, *navigational* memories.

Navigational information can be encoded using egocentric and/or allocentric spatial cues. In egocentric encoding, a location is encoded relative to the observer, while in allocentric encoding a location is encoded relative to external elements such as landmarks or boundaries^12–14^. While medial temporal lobe structures such as the hippocampus are strongly implicated in allocentric encoding^15–19^, egocentric encoding is supported by extra-hippocampal structures as the medial parietal cortex^17,20–22^, and importantly, can occur independently of the hippocampus^23,24^ (but see^14^). Notably, egocentric vs. allocentric neural systems are differentially vulnerable to normal aging processes^25–27^ with the hippocampus being among the first cerebral structures to show structural and functional changes in age^27^. Age-related neurodegeneration is also found in other brain areas that support navigation, as the prefrontal cortex^28^, which involved in navigational abilities of both place learning and response learning^29,30^. Accordingly, we aimed to determine whether the type of spatial encoding modulates the presumed age-related deficit in spatial updating.

Finally, it has been suggested that age-related inefficiency in memory updating occurs due to intrusions of former representations^31,32^. Specifically, age-related deficits in spatial updating were associated with elevated representations of the former spatial context^5,10^. Accordingly, we further asked whether the presumed age-related deficit in the ability to update navigational memories is associated with an elevated representation of the former navigational experience.

**Figure 1:**
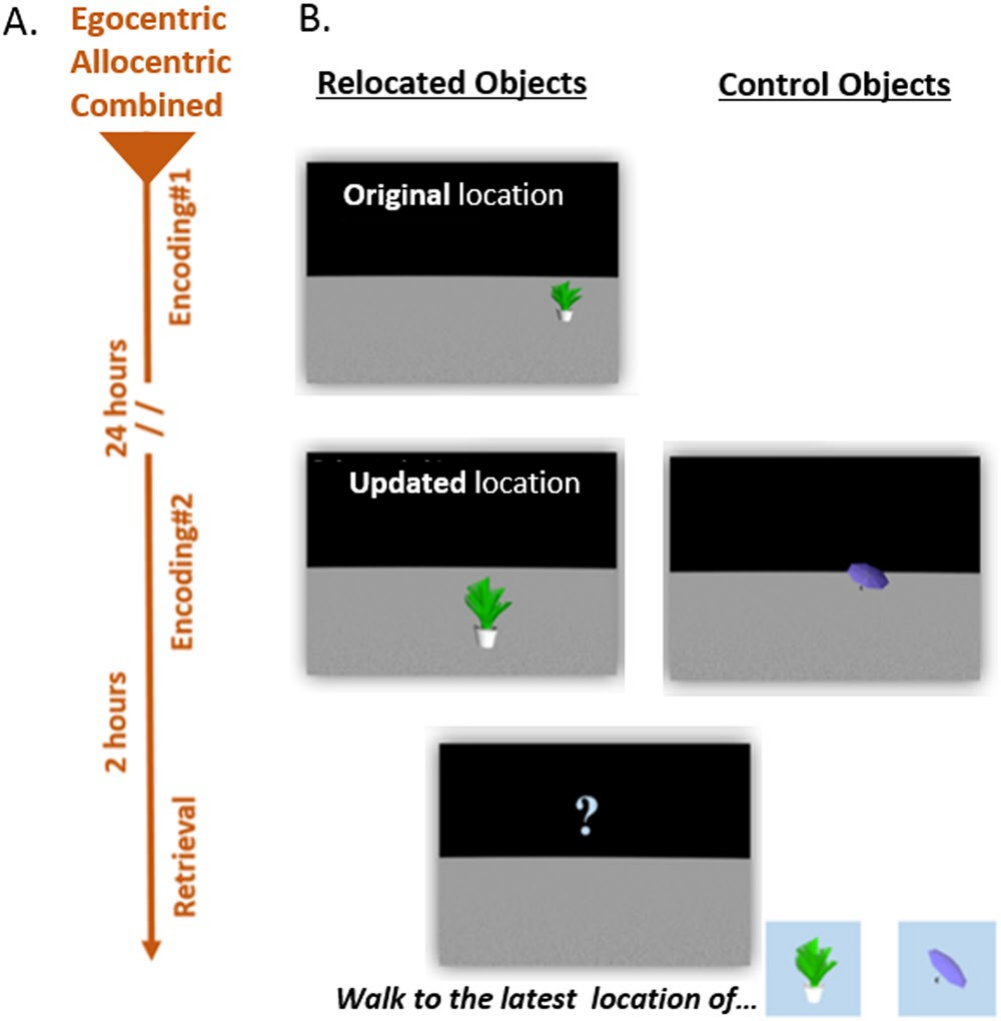
Experimental structure: (**A**) Timeline of the experiment. Every participant experienced three experimental sessions: encoding#1, encoding#2, and retrieval. Encoding#2 took place 24 hours after encoding#1 and retrieval took place two hours after the end of encoding#2. (**B**) Relocated and Control objects. Ten objects were presented at encoding#1, one at a time, each assigned to a specific location (top). At encoding#2, the ten objects from encoding#1 were presented in a different set of locations and thus served as the relocated objects (e.g., the plant) (**B**, middle, left). In addition, during encoding#2, ten objects that did not appear on encoding#1 were presented and served as the control (non-relocated) objects (e.g., the umbrella) (**B**, middle, right). During the retrieval session, participants were asked to walk to the *latest* location of each object (both for relocated and control objects) (**B**, middle, bottom).

**Figure 2:**
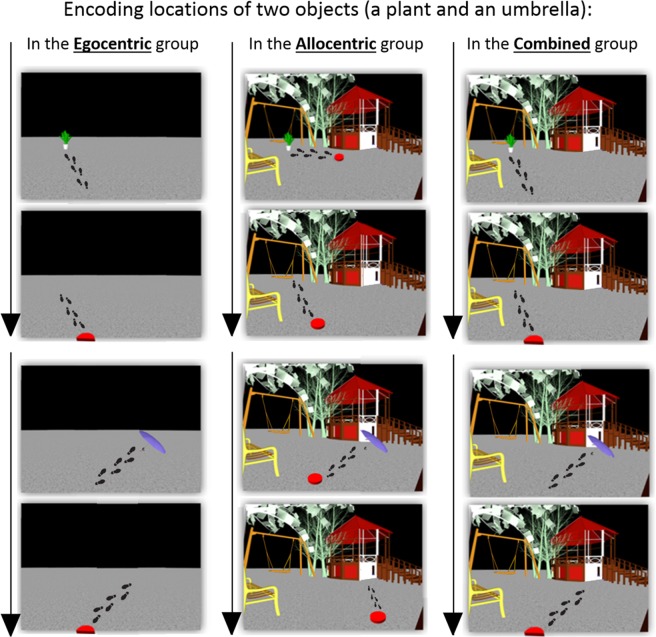
The three spatial-cue conditions. Participants were assigned to either the egocentric, the allocentric, or the combined condition. The footsteps (not shown in the task) represent exemplary paths that participants walked in each condition, to encode the locations of two objects (a plant and an umbrella). Left: The *egocentric condition* contained no spatial landmarks, and the distance and direction to each object were encoded from a fixed viewpoint (the red semicircle). On every trial, participants started at the red semicircle, walked to the object, and made a perceptual judgment. The object then disappeared, and participants walked back to the red semicircle. Next, a new object was shown, and the procedure was repeated. See video 1 for further details. Middle: In the *allocentric condition*, no fixed viewpoint was used as different viewpoints were used. On every trial, participants started at a temporary red circle, walked to the object, and made a perceptual judgment. The object then disappeared, and participants walked to another red circle (which appeared in a different location than before). However, participants could code the locations of the objects relative to environmental landmarks. Next, a new object was shown, and the procedure was repeated. See video 2 for further details. Right: In the *combined condition* (right), the locations of the objects could be coded relative to the fixed viewpoint and relative to the spatial landmarks. See video 3 for further details.

To address these questions, healthy younger and older participants learned the locations of objects in a fully immersive, three-dimensional virtual environment in two learning sessions, which took place on two consecutive days (Fig. 1A). Half of the objects were relocated overnight, and, eventually, participants had to indicate the latest locations of the relocated and the non-relocated (control) objects (Fig. 1B). The immersive virtual reality methodology allowed us to dissociate egocentric and allocentric spatial cues and to assess encoding and retrieval in each navigational strategy (Fig. 2 and videos 1–3). Specifically, in the egocentric group, participants could encode the location of the object only by the distances and directions relative to themselves, while, in the allocentric group, participants could encode the locations of the objects only by the directions and distances relative to environmental landmarks (Fig. 2). To assess whether the presumed spatial updating deficit in old age depends on a specific spatial cue or on age-related differences in the integration of egocentric and allocentric cues, we added a third condition in which both egocentric and allocentric cues were provided (i.e., the combined condition) (Fig. B). To assess the effect of the previous navigational context on spatial updating abilities, we measured the relative proximity of the behavioral responses to the original (pre-updated) locations. Finally, in experiment 2, we tested for age-related differences in overnight retention of navigational memories.

## Results

### Experiment 1

**Figure 3:**
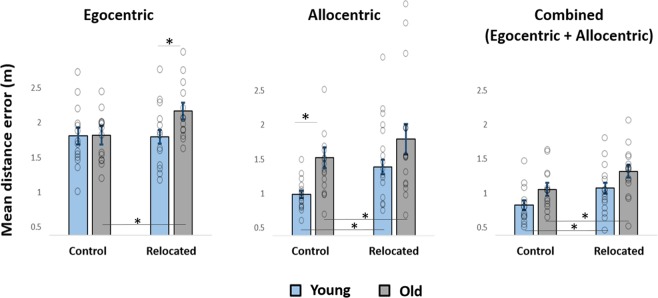
Age and spatial cues influence spatial memory and spatial updating. Distance errors to the latest locations of relocated and non-relocated (control) objects, in the egocentric (left) allocentric (middle) and combined (right) conditions, are shown. Shorter distance errors indicate higher retrieval accuracies. The control objects represent spatial memory while the difference between distance errors of relocated and control objects represent the spatial updating deficit. The *ANCOVA* revealed an age-related deficit in spatial updating in the egocentric condition. Data are presented as mean ± SEM. *p < 0.05 in post-hoc tests.

The aims of experiment 1 were to test whether the ability to update long-term, navigational memories is impaired in older adults and to explore the effect of different spatial cues on spatial updating abilities. Towards this, distance errors were analyzed in a mixed model ANCOVA, with object-type (control vs. relocated objects) as the within-subject factor and age and spatial cues as between-subject factors (Fig. 3). To control for potential age-related differences in the speed of walking, we added mean encoding duration as a covariate.

The analysis revealed a main effect of age, with larger distance errors for older adults (F (1, 81) = 8.05, p = 0.006, ƞp^2^ = 0.09). There was also a main effect of spatial cues (F (1, 81) = 28.79, p < 0.001, ƞp^2^ = 0.415). Post-hoc Bonferroni corrected multiple comparisons revealed that distance errors in the egocentric condition were larger than in the allocentric condition, and those in the allocentric condition were larger than in the combined condition (p < 0.005 in all three comparisons). The superior retrieval accuracy in the combined condition suggests that participants successfully integrated egocentric and allocentric cues^33^.

The analysis revealed no significant effect of object-type (F (1, 81) = 0.04, p = 0.85, ƞp^2^ < 0.005), no significant object-type X spatial cues interaction (F (1, 81) = 1.36, p = 0.263, ƞp^2^ = 0.032), no object-type X age interaction (F (1, 81) = 1.26, p = 0.27, ƞp^2^ = 0.015) and no age X spatial cues interaction (F (1, 81) = 0.86, p = 0.43, ƞp^2^ = 0.021). However, we observed a significant object-type X age X spatial cues interaction (F (1, 81) = 9.05, p < 0.005, ƞp^2^ = 0.183), (Fig. 3).

#### Age and spatial cues modify navigational memory

Following the object-type X age X spatial cues interaction, we first tested for the effects of age and spatial cues on navigational memory by analyzing distance errors for the control objects (Fig. 3). These objects were associated with a single set of locations during the second encoding session and thus did not require spatial updating (Fig. 1B). Post-hoc Bonferroni corrected pairwise comparisons indicated that in the egocentric and combined conditions, young and older participants showed comparable distance errors, indicating similar navigational memory (F (1, 81) < 0.005, p = 0.99, ƞp^2^ < 0.005 and F (1, 81) = 1.32, p = 0.252, respectively). On the other hand, in the allocentric condition, the young participants showed superior navigational memory, with shorter distance errors (F (1, 81) = 15.98, p < 0.005), (Fig. 3, middle, control objects).

#### Age and spatial cues modify spatial updating abilities

The object-type X age X spatial cues interaction suggests that both age and the type of spatial cue influence spatial updating abilities. Post-hoc, Bonferroni corrected pairwise comparisons indicated that in the egocentric condition, while young participants revealed comparable distance errors for control and relocated objects (F (1, 81) < 0.005, p = 0.995), older adults showed larger distance errors for the relocated objects (F (1, 81) = 6.34, p = 0.014), (Fig. 3, left). Unlike in the egocentric condition, in the allocentric condition, both age groups showed larger distance errors for the relocated objects (F (1, 81) = 11.45, p = 0.001 and F (1, 81) = 5.26, p = 0.024, respectively) (Fig. 3, middle). Similarly, in the combined condition, both groups showed larger distance errors for the relocated objects (F (1, 81) = 4.1, p = 0.046 and F (1, 81) = 4.43, p = 0.039, respectively) (Fig. 3, right). Together, these results demonstrate a selective deficit of older adults to update navigational memories in the egocentric condition.

#### Older adults show stronger representations of the former navigational context

**Figure 4:**
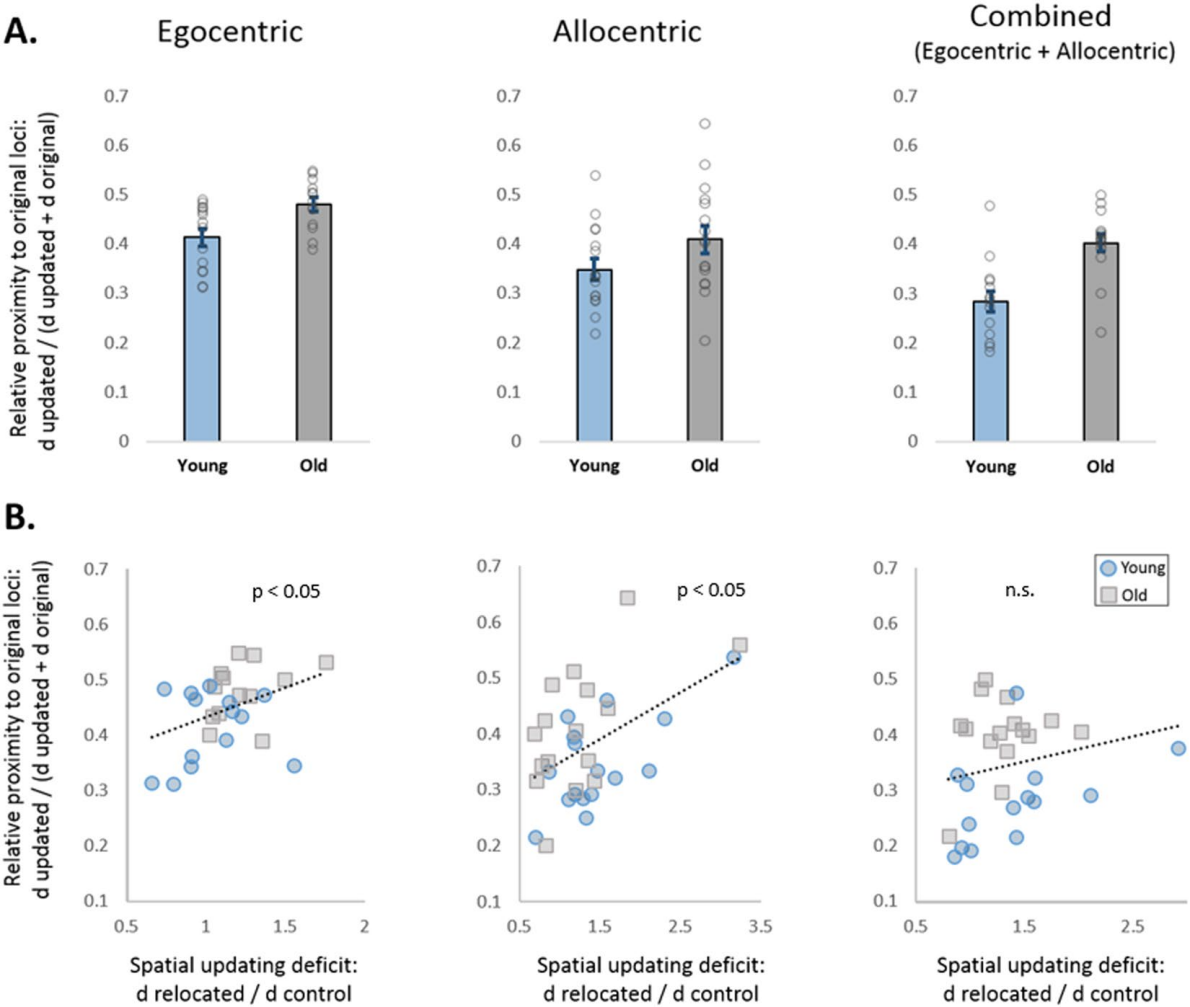
Older participants show stronger representations of the former navigational context, which are correlated with the spatial updating deficits. (**A**) Effects of the original locations on the retrieval of the updated ones. Higher bars indicate a greater effect of the original loci on the retrieval of the updated ones. Across conditions, this effect was found to be stronger in old age. (**B**) Spatial updating deficit is associated with stronger representations of the original navigational context. In the egocentric and the allocentric conditions, the effect of the original loci on retrieval of the updated ones was positively correlated with the spatial updating deficit. Data are presented as mean ± SEM.

In the previous section, we found that the spatial cues and age factors influence spatial updating abilities. To determine the source of the spatial updating deficit, we asked whether the former navigational context affects spatial representations of the novel navigational experience. Towards this, we measured the effect of the memory for the original locations on retrieval of the updated ones, which can be quantified by the relative proximity ($rProxOrig\,=\frac{1\,/\,dOrig}{1\,/\,dUpd+1\,/\,dOrig}=$ $\frac{dUpd}{dUpd+dOrig}$ See methods). A two-way ANCOVA with age and spatial cues as between-subjects factors revealed a main effect of age, with higher relative proximities to the original locations among older adults (F (1, 88) = 16.26, p < 0.005, ƞp^2^ = 0.17). This effect indicates a greater influence of the memories for the original locations on retrieval of the updated ones, among older adults (Fig. 4A). There was also a main effect of spatial cues (F (2, 87) = 11.51, p < 0.005, ƞp^2^ = 0.22). Post hoc, Bonferroni corrected multiple comparisons revealed that the relative proximity to the original locations in the egocentric condition was higher than in the other two conditions (allocentric: p < 0.01; combined: p < 0.001), (Fig. 4A), which depicted comparable relative proximities (p = 0.324). The analysis revealed no age X spatial cue interaction (F (2, 87) = 1.23, p = 0.30, ƞp^2^ = 0.03).

#### Memory representations of the former navigational experience are associated with spatial updating deficits

Next, we correlated the relative proximities to the original locations with the spatial updating deficits, defined as the ratio between the mean distance error to the *relocated* objects and the mean distance error to the *control* objects (d_relocated_/d_control_) (Fig. 4B). A linear regression analysis which included all the participants of experiment 1 revealed a significant, positive correlation (r = 0.26, n = 88, p = 0.014). To test whether this correlation was modified by age, spatial cues, or by age X spatial cues interaction, we ran a moderator analysis. The moderator analysis revealed that the spatial cue interaction term significantly increased the variation explained by 7% (p = 0.003), while the age and the age X spatial cues interaction terms did not (age: −4.5%, p = 0.143; age X spatial cues: −5.8%, p = 0.341).

Following the significant moderation effect of the spatial cues on the overall correlation, we ran additional regression analyses separately for each spatial cues condition. To minimize the effect of outlier responses, we applied robust correlation analyses. The analyses revealed positive correlations between the spatial updating deficit and the relative proximity to the original locations, in both the egocentric and allocentric conditions (r = 0.437, n = 27, p = 0.023 and r = 0.49, n = 32, p = 0.004, respectively), suggesting that traces of spatial representations from the original navigational context can hinder the updating of navigational memories (Fig. 4B). No significant correlation was found in the combined condition (r = 0.216, n = 29, p = 0.261). However, no significant difference was found between the correlation coefficient of the combined condition and the correlation coefficients of the egocentric and allocentric conditions (z = −0.69, p = 0.245; z = −1.26, p = 0.104, respectively).

### Experiment 2

In experiment 1, we found age-related deficits in spatial updating following egocentric encoding. We further found that the deficits in spatial updating were associated with stronger spatial representations of the original navigational context, which could be explained either by superior overnight retention or by inefficient suppression of the original navigational memories. Although previous studies have indicated that overnight consolidation is impaired in old age^34,35^, the aim of experiment 2 was to distinguish between both hypotheses by assessing overnight retention of egocentrically encoded spatial memories.

**Figure 5:**
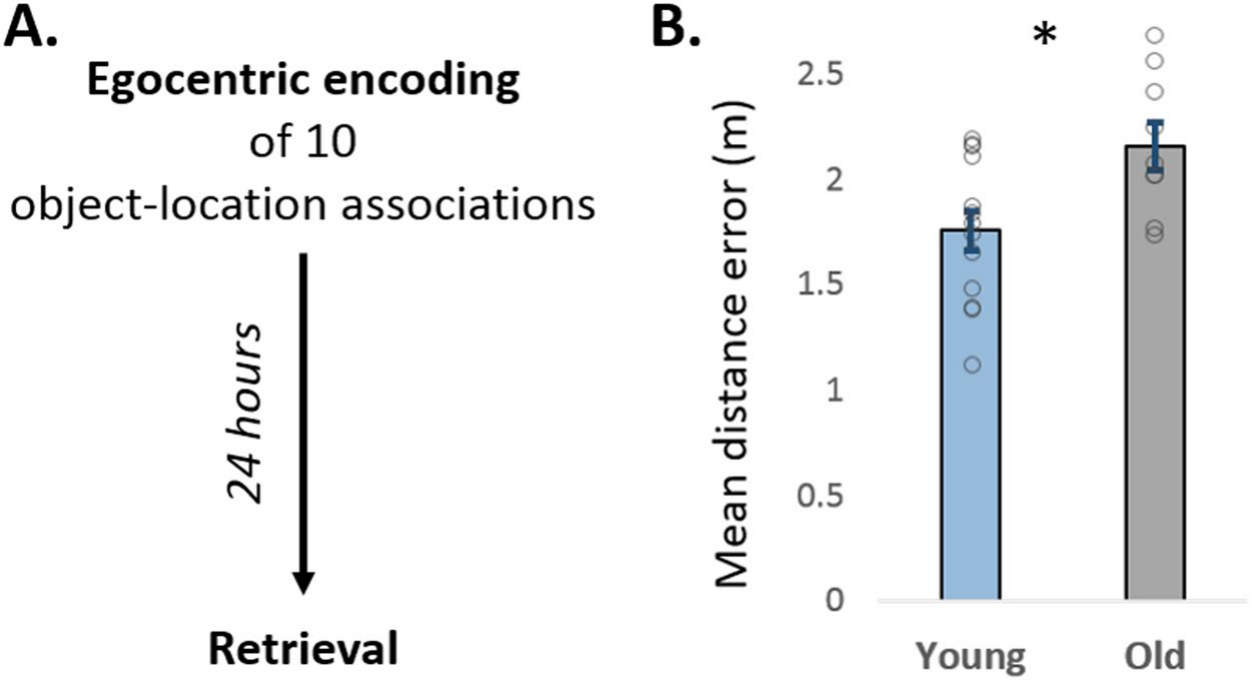
Overnight retention of navigational memories after egocentric encoding. (**A**) The procedure of experiment 2. Retrieval accuracy was assessed 24 hours after encoding in the egocentric condition. (**B**) Older adults showed larger distance errors, indicating inferior overnight retention of navigational memories. Data are presented as mean ± SEM.

To address this question, young and older adults had to retrieve locations of the objects learned on the previous day (Fig. 5A). The ANCOVA revealed that older adults showed larger distance errors than the young (F (1, 19) = 10.98, p = 0.004, ƞp^2^ = 3.7), depicting lower retrieval accuracy (Fig. 5B) and thus, inferior overnight retention. Importantly, this finding is in line with the notion of reduced overnight consolidation in aging^34,35^. Accordingly, we suggest that the stronger influence of the original navigational experience among the older adults in Experiment 1 cannot be explained by superior retention of the original navigational context but may rather stem from an inefficient suppression of the former spatial context.

## Discussion

The current study reveals age-related deficits in the ability to update navigational memories and thus proposes a mechanism for the navigational decline in human aging^2,36^. We found that the deficit in updating navigational memories in older adults was selective to the egocentric navigational condition. Spatial learning and memory often show substantial inter-individual differences, and particularly in aging. However, in the egocentric condition, the young and the older adults revealed comparable retrieval performance of control objects. The similarity in spatial learning between the two age groups suggests that in the egocentric condition, the age-related differences in the ability to retrieve the updated navigational memories are selectively due to age-related alternations in spatial updating abilities. Notably, while previous studies of egocentric encoding have largely focused on categorical knowledge (i.e., route decisions)^25,37,38^, the current study tested for quantitative, vectorial, egocentric knowledge. Additionally, the impaired ability to update navigational memories was associated with stronger spatial representations of the former navigational experience.

Both in the allocentric and the combined conditions, spatial memory in older adults, as measured by the control objects, was inferior to the spatial memory in the young. In contrast, older adults showed similar performance in the egocentric condition. This pattern of results is in line with previous findings which indicate that aging severely affects allocentric coding but leaves egocentric navigation relatively intact^8,26,37,39–42^. The innovation of the current study is that our experimental approach enabled us to directly measure fine-grained vectorial egocentric knowledge, whereas previous studies probed for categorical knowledge or stimulus-response associations (i.e., route decisions). Accordingly, we could compare metric measurements of navigational behavior between the different types of encoding strategies.

Another effect of spatial cues on spatial memory among the young participants was that retrieval of control objects in the egocentric condition was inferior to the retrieval of control objects in the allocentric condition. This effect is likely driven by the specifics of the environmental layout and the design of the current study, including the number of environmental elements used in the allocentric condition and the location of the fixed viewpoint in the egocentric condition. We thus predict that reducing the amount of the environmental elements and shifting the position of the fixed starting viewpoint would reverse the superior spatial memory following allocentric encoding in young participants.

In the current study, the age-related deficit in spatial updating was selective to the egocentric condition. This selectivity indicates that the encoding procedure may influence susceptibility to proactive interference among older adults. A similar effect of encoding procedure on the susceptibility to proactive interference in old age was found in semantic learning^43^. In that study, age-related proactive interference in long-term, semantic associations was selective to a semantic acquisition process, which, like egocentric encoding, can trigger a hippocampal-independent learning mechanism^43,44^ (but see^45,46^). Together, these findings suggest that there may be specific neural processes which underlie the inefficient updating of long term memories in older adults.

In line with previous findings^5,10^, older participants depicted stronger memory traces of the former navigational context (i.e., the original locations). Furthermore, the deficits in the ability to update spatial memories were associated with elevated representations of the previous navigational experience. Elevated representations of the former navigational context in old age can be explained either by inefficient suppression or by superior memory of the former navigational experience. Importantly, the retrieval accuracies of control objects in the egocentric condition (Fig. 3, left, control objects) indicate comparable encoding and short-term retention of the spatial information, between the young and the older adults. Furthermore, the results of experiment 2 suggest that overnight retention of the original spatial context is not superior among older adults. Together with former findings of age-related reduction in overnight (sleep-dependent) consolidation^34,35^, our findings argue against the explanation that the elevated representations of the former navigational context in old age are due to superior memory for the previous navigational experience. Instead, we propose that the stronger influence of the former navigational experience in old age is related to inefficient suppression of memory traces from the former navigational context and those memory traces may underlie the increased proactive-interference in old age^4,31,32,47^.

Several mechanisms have been implicated in the resolution of long-term, proactive interference and thus, in the ability to update long-term memories. One mechanism is modulation of cholinergic levels^48–50^ and particularly, the levels of cholinergic input from the basal forebrain to the hippocampus^51,52^. Age-related reduction in hippocampal responsivity to acetylcholine^53,54^ (for review, see^55^) may underlie the spatial updating deficits in old age. Another mechanism is dopamine-mediated synaptic plasticity in the CA1 hippocampal subfield when novelty is detected^56–58^. Age-related reduction in the number of dopamine receptors in CA1^59,60^ might contribute to impairments in synaptic plasticity and novelty detection, which are essential for efficient updating. Nevertheless, why were the spatial updating deficits in older adults selective to the egocentric condition? While it is conceivable that age-related changes in hippocampal processing might predominantly affect the resolution of proactive interference during egocentric encoding, this hypothesis awaits rigorous experimental testing.

## Methods

### Experiment 1

#### Participants

48 young and 48 older participants were recruited for experiment 1. In the egocentric encoding condition, there were 16 young (mean age: 24.19 ± 0.72 years, eight women) and 16 old (mean age: 71.5 ± 1.0 years, eight women) participants. In the allocentric encoding condition, there were 16 young (mean age 24 ± 0.8, eight women) and 16 old (mean age: 71.3 ± 1.1, eight women) participants. In the combined encoding condition, there were 16 young (mean age: 23.94 ± 0.8, eight women) and 16 old (mean age: 71.8 ± 1.5, eight women) participants.

Participants were paid €6.50 per hour and gave written informed consent to the experimental protocol, which was approved by the ethics committee of the Otto-von-Guericke-University of Magdeburg, Germany. All methods were performed in accordance with the relevant guidelines and regulations. All participants performed the Montreal Cognitive Assessment (MoCA^61^) to screen for mild cognitive impairment (lower than 23^62^) and the divided-attention test of the test-battery of attentional performance (TAP^63^) to screen for attentional deficits (more than 16% “missed” trials). Four young participants (two from the egocentric condition and two from the combined condition) and four older participants (three from the egocentric condition and one from the combined condition) were excluded from the experiment due to poor attentional performance.

#### Stimuli and apparatus

The experiment took place in the motion tracking laboratory of the DZNE Magdeburg. In all experimental phases, participants were “immersed” into a virtual reality (VR) scene, using a head-mounted display (HMD) (Fig. 1C). In order to navigate in the virtual environment, the location and orientation of the participants were continuously tracked using a high-resolution motion tracking system, which seamlessly updated the virtual environment as the user moved around. The stimuli of the experiment were two virtual environments designed using Vizard 5 (WorldViz) and 3ds Max (Autodesk) software, and twenty 3-dimensional, everyday virtual objects designed using 3ds Max. Each object appeared in a specific coordinate within the environment, forming an object-location association.

The virtual environments and the objects were presented in Vizard and displayed using the Oculus DK 2 HMD (refresh rate: 75 Hz), with a 100° nominal field of view and pixel resolution of 960 × 1080 per eye. The dimensions of the motion-tracking laboratory were 6 m x 10 m, while the motion detection area in which the participants navigated was 4.5 m × 7 m. The participant’s position was tracked via the Vicon Tracker, and the participant’s orientation was tracked via the internal sensor of the Oculus. The displayed image was updated accordingly. The slow angular drifting in the Oculus’s internal sensors was manually reset every four trials. To ensure that participants relied on the reference frames of the virtual environments, they were disoriented with respect to the actual physical dimensions of the motion tracking-laboratory. Towards this goal, before each immersive VR session, participants donned the HMD and were then moved and rotated on a rotating chair. In addition, to mask auditory cues that could be used to maintain orientation, participants heard a water-stream sound via headphones.

#### Experimental design and procedure

The experiment comprised two between-subjects factors of ‘age’ (young, old) and ‘spatial cues’ (egocentric; allocentric; combined) and one within-subjects factor of ‘object-type’ (control, relocated). The experiment took place over two consecutive days and comprised three sessions: ‘Encoding 1’, ‘Encoding 2’ and ‘Retrieval’ (Fig. 1A). ‘Encoding 1’ took place on the first day. In this session, participants were presented with ten object-location associations, one at a time (Fig. 1B). The presentation of the ten object-location associations was repeated three times in different pseudorandom orders.

‘Encoding 2’ took place on the second day, during which 20 object-location associations were presented, one at a time (Fig. 1B). Ten of the twenty associations presented at ‘Encoding 2’ contained the objects that were shown during ‘Encoding 1’, but in a *different* set of locations than in ‘Encoding 1’. These were the relocated objects (Fig. 1B). The other ten objects presented at ‘Encoding 2’ were novel and served as the control objects (Fig. 1B). There was no overlap between the 20 loci of ‘Encoding 2’ and the ten loci of ‘Encoding 1’. The presentation of the 20 associations at ‘Encoding 2’ was repeated three times in different pseudorandom orders. The control and relocated object-location associations were counterbalanced between participants, to avoid a mnemonic bias toward specific objects and/or locations. The mean distance from the original (previous) to the updated (new) loci of the relocated objects was 3.18 ± 0.12 m (min = 2.26 m, max = 4.08 m), and was similar across the two counterbalanced sets of object-location associations (t (22) = 0.47, p = 0.64).

Since incidental encoding may enhance updating deficits in old age^10,43^, the object-location associations were incidentally encoded. Towards this, participants were not informed that the task involves mnemonic skills; instead, they were told that the task assesses age-related differences in perceptual judgment over time and repetitions. Accordingly, in each encoding trial, participants were asked to walk directly towards the object and to report the number of its colors. Additionally, as participants should have not intentionally encode the locations of the objects, they had to be naïve regarding the purpose of the task. Therefore, the spatial cue factor was between, rather than within participants.

Retrieval took place on the 2^nd^ day, two hours after ‘Encoding 2’ (Fig. 1A). On each retrieval trial, participants were first presented with a picture of one of the 20 objects from ‘Encoding 2’, for five seconds (Fig. 1B, bottom). Then, participants had to walk to the location in the environment in which that object had appeared *last* (i.e., 2 hours earlier, at ‘Encoding 2’). This location will be referred to as the updated location. In case participants did not remember the location, they were asked to guess. Retrieval accuracy of the control and the relocated objects was quantified as the mean Euclidean distance from the behavioral responses to the correct locations.

#### Manipulation of spatial cues

The virtual environment of the *egocentric condition* contained no elements except an “endless” concrete-like ground and a dark-grey background (Fig. 2, left). To ensure egocentric encoding, i.e., that locations were coded according to the distance and angle relative to the observer, all object-location associations were learned (and also retrieved) from the same location and orientation. Towards this, before each encoding and retrieval trial, the participant had to walk to a fixed viewpoint, marked by a red semicircle (Fig. 2, left), to stand behind its straight line and face its curved line. Once the participant had reached the specific location (within a 30 cm radius) and orientation (±15°), the red semicircle disappeared, and the to-be-encoded object appeared. Then, the participant had to walk directly to the object and to report the number of its colors. When the participant reached the object, it disappeared, and the red semicircle reappeared (in the same location and orientation, generating the same viewpoint), so the participant could return to it. Then again, as the participant was in the fixed viewpoint, the next to-be-encoded object appeared in its defined location (Fig. 2, left, bottom). Importantly, the same fixed viewpoint was used in both encoding sessions and also in the retrieval session. Encoding locations of two objects in the egocentric condition is represented in video 1.

In the *allocentric condition*, participants had to code and retrieve the locations of the objects only in relation to environmental landmarks. Therefore, in addition to the ground and background of the egocentric condition, the virtual environment of the allocentric condition contained several landmarks (Fig. 2, middle) that surrounded the area wherein the objects were presented. To make the encoding and retrieval experience of the allocentric condition comparable to those of the egocentric condition, participants had to walk to a ‘transient’ viewpoint before each encoding (or retrieval) trial (Fig. 2, middle). However, unlike the fixed viewpoint of the egocentric condition, the locations of the viewpoints in the allocentric condition *changed* between the trials. Importantly, to avoid consistent egocentric information regarding the location of an object, different viewpoints were used across the three encoding repetitions of each object-location association (Fig. 2, middle-bottom) and, particularly, between the retrieval trial and the encoding trials of each object. As in the encoding of the egocentric condition, participants had to walk to each object and to report the number of its colors. When participants reached the object, it disappeared, and a new viewpoint appeared. Once the participant had reached the next viewpoint, the next to-be-encoded object appeared in its defined location (Fig. 2, middle-bottom). Encoding locations of two objects in the allocentric condition is represented in video 2.

Finally, in the *combined condition*, both egocentric and allocentric cues were provided (Fig. 2, right). Specifically, participants could code and retrieve the locations of the objects both relative to their location and relative to environmental landmarks. Towards this goal, during all encoding and retrieval trials, we used the fixed viewpoint, as in the egocentric condition, together with the visual input of the allocentric condition (Fig. 2, right). Thus, participants could use both egocentric and allocentric cues to code and retrieve the locations of the objects. Encoding locations of two objects in the combined condition is represented in video 3.

#### Statistical analyses

The primary dependent variable in the study was the mean distance error, quantified by the mean Euclidean distance between the response and the correct location. Shorter distance errors indicate superior retrieval accuracy. A second dependent variable was ‘spatial updating deficit,’ which was defined by the mean distance to relocated objects divided by the mean distance to the control objects (d_upd_/d_con_). In addition, we calculated the ‘relative proximity to the original location’ (rProxOrig). If the distance to the updated location is dUpd and distance to the original location is dOrig (Fig. 2A), then the relative proximity (i.e., 1/distance) to the original location (rproxOrig) is $rProxOrig\,=\frac{1\,/\,dOrig}{1\,/\,dUpd+1\,/\,dOrig}=\frac{dUpd}{dUpd+dOrig}$^64^. Thus, higher relative proximity to the original location (rProxOrig) reflects a stronger effect of the original locations on the retrieval of the updated ones. If the distance to the updated and the original location is equal, then rProxOrig = 0.5. If the distance to the original location is larger than the distance to the updated location (i.e., the responses fall closer to the updated loci), then 0 < rProxOrig < 0.5. Finally, if the distance to the original location is shorter than the distance to the updated location (i.e., the responses fall closer to the original loci), then 0.5 < rProxOrig < 1. Last, to control for age differences in walking speed, we added a covariate of mean encoding duration.

SPSS (IBM SPSS Statistics 21) was used for analyses of variances and covariance (ANCOVA) of multifactorial effects. Significant effects were followed by Bonferroni corrected post-hoc tests. Robust linear regressions were analyzed using the rlm function in R (version 3.0.2).

### Experiment 2

Thirteen young (mean age: 23.2 ± 1.1 years, six women) and nine older adults (mean age: 73.2 ± 1.1 years, five women) participated in experiment 2. The participants were paid €6.50 per hour and gave a written informed consent to the experimental protocol, which was approved by the ethics committee of the Otto-von-Guericke-University of Magdeburg, Germany. All methods were performed in accordance with the relevant guidelines and regulations. As in experiment 1, the participants performed the Montreal Cognitive Assessment (MoCA^61^) and the divided-attention test of the test-battery of attentional performance (TAP^63^) to screen for mild cognitive impairment and attentional deficits, respectively.

The task took place over two consecutive days and consisted of two sessions; an encoding session on day 1 and a retrieval session on day 2 (Fig. 5A). Thus, experiment 2 did not comprise an ‘Encoding 2’ session. The encoding session was identical to the ‘Encoding 1’ session of the egocentric condition of experiment 1, in which participants were presented with the ten object-location associations. Retrieval took place the next day, using the same retrieval procedure that was used in the egocentric condition of experiment 1. However, here participants were asked to indicate the locations of the ten objects, as presented on day 1 (Fig. 5A). Overnight retrieval performance was compared between young and older participants using a two-tailed t-test.

## Supplementary information

Video 1

Video 2

Video 3

## References

[1] Coutrot A (2018). Global Determinants of Navigation Ability. Current biology: CB.

[2] Lester AW, Moffat SD, Wiener JM, Barnes CA, Wolbers T (2017). The Aging Navigational System. Neuron.

[3] Moffat SD, Resnick SM (2002). Effects of age on virtual environment place navigation and allocentric cognitive mapping. Behav Neurosci.

[4] Wilson IA, Gallagher M, Eichenbaum H, Tanila H (2006). Neurocognitive aging: prior memories hinder new hippocampal encoding. Trends in neurosciences.

[5] Wilson IA (2004). Cognitive aging and the hippocampus: how old rats represent new environments. Journal of Neuroscience.

[6] Adamo DE, Briceno EM, Sindone JA, Alexander NB, Moffat SD (2012). Age differences in virtual environment and real world path integration. Frontiers in aging neuroscience.

[7] Allen GL, Kirasic KC, Rashotte MA, Haun DB (2004). Aging and path integration skill: kinesthetic and vestibular contributions to wayfinding. Perception & psychophysics.

[8] Harris MA, Wolbers T (2012). Ageing effects on path integration and landmark navigation. Hippocampus.

[9] Stangl M (2018). Compromised Grid-Cell-like Representations in Old Age as a Key Mechanism to Explain Age-Related Navigational Deficits. Current biology: CB.

[10] Merhav M, Riemer M, Wolbers T (2019). Spatial updating deficits in human aging are associated with traces of former memory representations. Neurobiol Aging.

[11] Hegarty M, Montello DR, Richardson AE, Ishikawa T, Lovelace K (2006). Spatial abilities at different scales: Individual differences in aptitude-test performance and spatial-layout learning. Intelligence.

[12] Paillard, J. Motor and representational framing of space. *Brain and space*, 163–182 (1991).

[13] Pani JR, Dupree D (1994). Spatial reference systems in the comprehension of rotational motion. Perception.

[14] Wang R, Spelke E (2002). Human spatial representation: insights from animals. Trends in cognitive sciences.

[15] Compton DM (2004). Behavior strategy learning in rat: effects of lesions of the dorsal striatum or dorsal hippocampus. Behavioural processes.

[16] Ekstrom AD (2003). Cellular networks underlying human spatial navigation. Nature.

[17] Galati G (2000). The neural basis of egocentric and allocentric coding of space in humans: a functional magnetic resonance study. Experimental brain research.

[18] Hartley, T., King, J. A. & Burgess, N. Studies of the neural basis of human navigation and memory. *The neurobiology of spatial behavior. Oxford University Press, New York*, 144–164 (2003).

[19] O’Keefe, J. In *Progress in brain research* Vol. 83 301–312 (Elsevier, 1990). 10.1016/s0079-6123(08)61258-3 2203101

[20] Byrne P, Becker S, Burgess N (2007). Remembering the past and imagining the future: a neural model of spatial memory and imagery. Psychological review.

[21] Schindler A, Bartels A (2013). Parietal cortex codes for egocentric space beyond the field of view. Current Biology.

[22] Vallar G (1999). A fronto-parietal system for computing the egocentric spatial frame of reference in humans. Experimental brain research.

[23] Feigenbaum JD, Rolls ET (1991). Allocentric and egocentric spatial information processing in the hippocampal formation of the behaving primate. Psychobiology.

[24] Goodrich‐Hunsaker NJ, Livingstone SA, Skelton RW, Hopkins RO (2010). Spatial deficits in a virtual water maze in amnesic participants with hippocampal damage. Hippocampus.

[25] Iachini T, Ruggiero G, Bartolo A, Rapuano M, Ruotolo F (2019). The Effect of Body-Related Stimuli on Mental Rotation in. Children, Young and Elderly Adults. Scientific reports.

[26] Moffat SD, Resnick SM (2002). Effects of age on virtual environment place navigation and allocentric cognitive mapping. Behavioral neuroscience.

[27] Raz, N. Aging is a manifold of universal biological processes that, with passage of time, profoundly alter anatomy, neurochemistry, and physiology of all organisms. Although no organs or systems escape the impact of aging, its effects on the central nervous system (CNS) are especially dramatic. The brains of older people can be distinguished from those of their younger peers in many ways and on many levels, from mitochondria to gross anatomy. So numerous and diverse are the changes that encompassing the totality of brain aging in one survey would be too daunting an objective. Thus, for comprehensive up-to-date accounts of neurobiology. *Cognitive Neuroscience of Aging: Linking Cognitive and Cerebral Aging: Linking Cognitive and Cerebral Aging*, **19** (2004).

[28] Moffat SD, Kennedy KM, Rodrigue KM, Raz N (2007). Extrahippocampal contributions to age differences in human spatial navigation. Cereb Cortex.

[29] De Bruin J, Swinkels W, De Brabander J (1997). Response learning of rats in a Morris water maze: Involvement of the medial prefrontal cortex. Behavioural brain research.

[30] de Bruin JP, Moita MP, de Brabander HM, Joosten RN (2001). Place and response learning of rats in a Morris water maze: differential effects of fimbria fornix and medial prefrontal cortex lesions. Neurobiology of learning and memory.

[31] Hasher L, Chung C, May CP, Foong N (2002). Age, time of testing, and proactive interference. Canadian Journal of Experimental Psychology/Revue canadienne de psychologie expérimentale.

[32] May CP, Hasher L (1998). Synchrony effects in inhibitory control over thought and action. Journal of Experimental Psychology: Human Perception and Performance.

[33] Bates SL, Wolbers T (2014). How cognitive aging affects multisensory integration of navigational cues. Neurobiology of aging.

[34] Scullin MKS (2013). memory, and aging: the link between slow-wave sleep and episodic memory changes from younger to older adults. Psychol Aging.

[35] Harand C (2012). How aging affects sleep-dependent memory consolidation?. Front Neurol.

[36] Moffat SD (2009). Aging and spatial navigation: what do we know and where do we go?. Neuropsychology review.

[37] Wiener JM, de Condappa O, Harris MA, Wolbers T (2013). Maladaptive bias for extrahippocampal navigation strategies in aging humans. Journal of Neuroscience.

[38] Wolbers T, Weiller C, Büchel C (2004). Neural foundations of emerging route knowledge in complex spatial environments. Cognitive Brain Research.

[39] Gazova I (2013). Spatial navigation in young versus older adults. Frontiers in aging neuroscience.

[40] Iaria G, Palermo L, Committeri G, Barton JJ (2009). Age differences in the formation and use of cognitive maps. Behavioural brain research.

[41] Moffat SD, Elkins W, Resnick SM (2006). Age differences in the neural systems supporting human allocentric spatial navigation. Neurobiology of aging.

[42] Rosenbaum RS, Winocur G, Binns MA, Moscovitch M (2012). Remote spatial memory in aging: all is not lost. Frontiers in aging neuroscience.

[43] Merhav M, Karni A, Gilboa A (2014). Neocortical catastrophic interference in healthy and amnesic adults: a paradoxical matter of time. Hippocampus.

[44] Sharon T, Moscovitch M, Gilboa A (2011). Rapid neocortical acquisition of long-term arbitrary associations independent of the hippocampus. Proceedings of the National Academy of Sciences.

[45] Smith CN, Urgolites ZJ, Hopkins RO, Squire LR (2014). Comparison of explicit and incidental learning strategies in memory-impaired patients. Proceedings of the National Academy of Sciences.

[46] Warren DE, Duff MC (2014). Not so fast: Hippocampal amnesia slows word learning despite successful fast mapping. Hippocampus.

[47] Hasher, L., Zacks, R. T. & May, C. P. Inhibitory control, circadian arousal, and age (1999).

[48] Atri A (2004). Blockade of central cholinergic receptors impairs new learning and increases proactive interference in a word paired-associate memory task. Behav Neurosci.

[49] Hasselmo ME (2006). The role of acetylcholine in learning and memory. Current opinion in neurobiology.

[50] Hasselmo ME, Bower JM (1993). Acetylcholine and memory. Trends in neurosciences.

[51] De Rosa E, Hasselmo ME (2000). Muscarinic cholinergic neuromodulation reduces proactive interference between stored odor memories during associative learning in rats. Behavioral neuroscience.

[52] De Rosa E, Hasselmo ME, Baxter MG (2001). Contribution of the cholinergic basal forebrain to proactive interference from stored odor memories during associative learning in rats. Behavioral neuroscience.

[53] Haigler H, Cahill L, Crager M, Charles E (1986). Acetylcholine, aging and anatomy: differential effects in the hippocampus. Brain research.

[54] Lippa AS (1980). Brain cholinergic dysfunction and memory in aged rats. Neurobiology of Aging.

[55] Decker MW (1987). The effects of aging on hippocampal and cortical projections of the forebrain cholinergic system. Brain Research Reviews.

[56] Lisman JE, Grace AA (2005). The hippocampal-VTA loop: controlling the entry of information into long-term memory. Neuron.

[57] Lisman JE, Otmakhova NA (2001). Storage, recall, and novelty detection of sequences by the hippocampus: elaborating on the SOCRATIC model to account for normal and aberrant effects of dopamine. Hippocampus.

[58] Otmakhova NA, Lisman JE (1999). Dopamine selectively inhibits the direct cortical pathway to the CA1 hippocampal region. Journal of Neuroscience.

[59] Hemby SE, Trojanowski JQ, Ginsberg SD (2003). Neuron‐specific age‐related decreases in dopamine receptor subtype mRNAs. Journal of Comparative Neurology.

[60] Kaasinen V (2000). Age-related dopamine D2/D3 receptor loss in extrastriatal regions of the human brain. Neurobiology of aging.

[61] Nasreddine ZS (2005). The Montreal Cognitive Assessment, MoCA: a brief screening tool for mild cognitive impairment. Journal of the American Geriatrics Society.

[62] Luis CA, Keegan AP, Mullan M (2009). Cross validation of the Montreal Cognitive Assessment in community dwelling older adults residing in the Southeastern US. International journal of geriatric psychiatry.

[63] Zimmermann, P. & Fimm, B. A test battery for attentional performance. *Applied neuropsychology of attention. Theory, diagnosis and rehabilitation*, 110–151 (2002).

[64] Nardini M, Jones P, Bedford R, Braddick O (2008). Development of cue integration in human navigation. Current biology.

